# Editorial: Liquid biopsy: A tool for better understanding of the metastatic process ecosystem

**DOI:** 10.3389/fonc.2022.951158

**Published:** 2022-08-10

**Authors:** Matteo Bocci, Francesco Fabbri, Rui P.L. Neves, Elisabetta Rossi

**Affiliations:** ^1^ Department of Laboratory Medicine, Division of Translational Cancer Research, Lund University Cancer Centre, Medicon Village, Lund University, Lund, Sweden; ^2^ Biosciences Laboratory, IRCCS Istituto Romagnolo per lo Studio dei Tumori (IRST) “Dino Amadori”, Meldola, Italy; ^3^ Department of General, Visceral and Pediatric Surgery, University Hospital and Medical Faculty of the Heinrich-Heine University Düsseldorf, Düsseldorf, Germany; ^4^ Department of Surgery, Oncology and Gastroenterology, Oncology Section, University of Padua, Padua, Italy; ^5^ Veneto Institute of Oncology IOV-IRCCS, Padua, Italy

**Keywords:** liquid biopsy, metastatic cancer, cancer, tumor ecosystem, circulating tumor

Liquid biopsy is a non- or minimally-invasive procedure that allows retrieving analytes of different nature and different origins from body fluids including blood, urine, and cerebrospinal fluid (CSF). In the field of oncology, this sampling strategy has gained considerable attention and a growing number of technologies have been developed to inspect —in a fairly quick and economical way— circulating tumor cells (CTCs), extracellular vesicles (EVs), circulating-free tumor (ct) DNA, RNA, and a variety of non-coding RNAs from blood. Initially, these biomarkers have been gauged for their value as clinical indicators for diagnosis, prognosis as well as biomarkers for response to therapeutic regimens (1, 2). However, CTCs, ct nucleic acids, and EVs also play an active role in the metastatic process (3, 4), suggesting that liquid biopsy might also provide new opportunities to explore the biological and molecular mechanisms of tumor dissemination. On the one hand, CTCs have proven metastasis-initiating properties, indicating that these cells have the potential to inform on the molecular features necessary to reach and colonize distant organs. On the other hand, the ability of primary tumors to successfully spread to distant organs is dependent on interactions with microenvironments in distinct niches, and both CTCs (either alone, in clusters or aggregated with platelets/lymphocytes) and tumor-derived genetic material have been previously established as pivotal players in shaping such communication cues (5–8). Thus, the opportunity to detect blood-borne analytes should have a profound impact on our understanding of the systemic disease, and consequently on the management and monitoring of human malignancies.

This Research Topic comprises a series of 16 independent contributions addressing the potential utility of liquid biopsy as a complementary means to tackle specific, sorely needed, and unresolved clinical issues.

One such example comes from the inherent difficulty of detecting the presence of specific tumor types (9), which are often only diagnosed when they have already systemically spread in the organism. Xu et al. developed a prediction tool based on a regression model that integrates data from sequencing and epigenetic analysis of urine samples, and further identified the methylation status of the *ONECUT2* gene as a prospective biomarker for the detection of upper tract urinary carcinomas. With a similar goal in mind, Rikkert et al. reported on the efforts of a Dutch consortium to assess the available technologies for the recognition, classification, characterization and enumeration of the different types of tumor-derived (td)EVs in blood samples when using prostate cancer cell lines as a reference.

The availability of approved companion diagnostic tools is a reality for some tumor types with specific mutational patterns. Indeed, already in 2013, the Food and Drug Administration (FDA) agency approved the use of CellSearch for the detection of CTCs in metastatic breast, colorectal and prostate cancer (10). More recently, the same federal bureau cleared Foundation Medicine’s FoundationOne^®^Liquid CDx platform to monitor the response to standard of care by evaluating the rearrangements, mutations, single nucleotide variants (SNVs), and *Indels* in ctDNA in breast, prostate, ovarian and non-small cell lung cancer (NSCLC). In this special issue, two papers focus on EGFR mutations specifically in NSCLC: Nardo et al. performed a retrospective droplet qPCR analysis on co-existing *KRAS* alterations in EGFR-mutated NSCLC patients post-first-line tyrosine kinase inhibitor (TKI) treatment. Despite the low number of samples that will require further validation, low-frequency co-mutations seemed to reduce the efficacy of targeted EGFR therapy. In addition to such events, uncovering secondary mutations that might emerge *de novo* or through the selection of pre-existing clones is a fundamental aspect to offer patients the best available therapeutic option. In the context of this framework, Liu et al. presented the results of a pipeline which had a twofold purpose: first, to compare EGFR mutations in primary tissue and circulation (in ctDNA and CTCs); second, to develop a multimodal analysis of secondary mutational events from a single blood sample, to spare additional invasive sampling in patients.

However, the actual role of liquid biopsy-based protocols for early detection of lung cancer seems more nebulous: as highlighted in the review article by Freitas et al., sensitivity and specificity of current technologies are still the limiting factors for the ambitioned transition from research environments to clinical routines. These aspects become even more challenging for tumor types with scarce evidence-based literature: a case in point is the underlooked role of tdEVs in the biology of gynecological malignancies, which is the ground zero for the perspective paper by Herrero et al. In a similar fashion, taking advantage of the tumor-specific circulome is not up to par in clear cell renal cell carcinoma (ccRCC), with Lakshminarayanan et al. detailing how the current strategies have fallen short of the promised revolution.

Together with driver genetic mutations, additional factors contribute to the broad spectrum of progression and response to treatment. From a clinical standpoint, these biological components represent —if validated— attractive biomarkers with diagnostic, predictive or prognostic value. One interesting approach is described by Gu et al., as a specific tRNA-derived fragment seemed to bear diagnostic and post-operative capabilities in gastric cancer. Notably, this marker correlated with disease progression and showed a gradient expression allowing to distinguish healthy donors from patients affected by different pathologies of the stomach, including inflammation-driven gastritis and cancer. Furthermore, molecular profiling can aid the traditional histopathological classification of tumors. For example, breast cancer taxonomy is clinically very well defined, with a series of markers that guide treatment decisions, and are indicative of clinical performance, outcome, and risk of metastatic disease. As the name suggests, triple-negative breast cancer (TNBC) lacks the expression of three major drivers of the proliferation of the malignant compartment, namely hormone receptors (Estrogen and Progesterone) as well as the Human Epidermal growth factor receptor (HER)2. Therefore, these tumors are currently not eligible for targeted drugs, with chemo- and radiation therapy still representing the mainstay offer. Nonetheless, through gene expression profiling, a subset of the TNBC tumors is further characterized by an enrichment for an androgen receptor (AR) signature, suggesting a functional signalling that might be exploited for therapeutic gain. The study led by Kasimir-Bauer et al. determined that in primary non-metastatic TNBC, AR-related genes can be found in CTCs that are effectively wiped out by chemotherapy. However, these markers seemed to confer an intrinsic higher risk of early relapse as well as an association with reduced overall survival. Additionally, the authors noted that the CTCs also harbored a specific AR splice variant, which is known to be associated with therapeutic resistance.

In parallel, the above-mentioned obstacles curb the use of liquid biopsy for the early detection of metastatic disease. It is indeed conceivable that tumor-derived circulating components could be found at any given time throughout tumorigenesis, including metastatic disease. At the same time, it is important to stress that a curative intent is much more likely to succeed before systemic spread occurs (11). Eslami-S et al. authored a comprehensive review of the contribution of liquid biopsy analytes in every step of the metastatic cascade, thereby serving as a backbone for many of the original articles collected in this special issue. For instance, Gurioli et al. followed castration-resistant prostate cancer patients exposed to standard of care to assess the impact of copy number status for AR on clinical outcomes. Jiang et al. compared a panel of somatic mutations that affect pancreatic adenocarcinoma development and progression in primary tumors and plasma-borne ctDNA, and their results were further correlated to clinical parameters like tumor size, disease-free survival, and vascular invasion to predict recurrence.

Alongside these more established biomarkers, another set of papers proposes a broader range of prognostic markers based on circulating catabolites, micro (mi)RNAs as well as other hallmarks of cancer. In the context of metastatic prostate cancer, Sharova et al. aimed to identify blood-borne cell-free miRNAs that could predict the response to two different AR-targeting agents that are currently available for patients, while Crotti et al. employed mass spectrometry to determine the significance of tryptophan catabolism as a prognostic factor in locally advanced rectal cancer prior to preoperative chemotherapy. Interestingly, the authors found that tryptophan levels were differentially regulated in responsive *vs.* non-responsive patients. By expanding the analysis to the enzymes that are involved in the catabolism of this amino acid, discordant results were obtained between plasma and primary tissue, thus warranting further investigations. Finally, two studies shed light on capturing genetic events such as allelic imbalance and genomic instability. The former, led by Boldrin et al. used a panel of microsatellite and single nucleotide polymorphisms (SNPs) in blood as a readout for tumor burden during treatment in a cohort of patients diagnosed with locally advanced esophageal cancer. In agreement with the intended chief use of liquid-based screening, a longitudinal follow-up in a few selected patients further revealed that tumor-specific alterations could be detected in the circulation before symptomatic or overt clinical signs of relapse. In the latter, Wu et al. compared ctDNA in matched plasma and CSF from NSCLC patients with leptomeningeal metastases (LM). This work highlights the gain on sensitivity for mutation detection obtained by analysis of CSF and, intriguingly, this analysis revealed the enrichment of a specific mutation linked to acquired resistance to TKO inhibitors exclusively in the CSF, suggesting different mechanisms of cancer evolution between LM and extracranial lesions.

Even though the body of work presented in this collection focuses on pivotal aspects of liquid biopsy with a clear clinical application, outstanding questions remain unanswered. Among these is the overall issue of validation, as preliminary analyses are often performed in cohorts that are not sufficiently large to make solid and substantial claims from a statistical perspective. This goes hand in hand with the aforementioned specificity and sensitivity barrier of certain approaches. Nonetheless, it is also fundamental to acknowledge how dynamic the field of liquid biopsy has been, which is well reflected by the exponentially growing number of publications, particularly in the last decade. Importantly, the knowledge produced so far has resulted in great technological advances that are already reducing the gap between the bench and the bed, as highlighted before. Two of the key strengths of liquid biopsy are its power to inform about tumor features even when conventional tissue biopsy is not possible, and to shed light about residual disease long before overt clinical symptomatology. Indeed, this feature could be exploited in large-scale prevention screenings, *e.g.* together with mammographic examination for breast cancer or the fecal immunochemical test for colon cancer. Additionally, we believe that monitoring tumor *via* liquid biopsy will be an indispensable companion tool during treatment to offer more dynamic and proactive personalized regimens. Conceivably, this approach could minimize the risk of therapeutic resistance that afflicts many patients as the tumor progresses in an active state of selective pressure. An additional element central to the implementation of liquid biopsy is the notable increase in the quality and depth of sampling as the technology progresses, allowing for the selection of mutations with clear clinical relevance. This might overcome certain limitations peculiar to the early disease, a stage characterized by a limited concentration of blood-borne tumor-derived material. In this scenario, assays could be developed to identify and distinguish selective mutations rather than indiscriminately fishing out analytes without a clear clinical annotation that shed from primary tumors. Further promising assays are currently under development or awaiting clinical approval, including *e.g.* the “one tube fits all” CancerSEEK for multi-analytes for pan-cancer discovery (12), the SAGA Diagnostics’ SAGAsafe for the longitudinal monitoring of EGFRT790M mutations in lung cancer (13), or the Illumina’s TruSight Oncology 500 portfolio (14). These developments are good indicatives and we are positive about a gradually stronger impact of liquid biopsy in the clinics. In connection with the real implementation of liquid biopsy in the clinical setting, this procedure offers an unprecedented opportunity to sample the systemic disease and address questions related to the biology of cancer. One subject of paramount interest is whether this approach could help discriminate which organs are affected by metastatic colonization, as already suggested for EVs (15), thereby implying a differential composition of tumor-derived particles and catabolites dispersed in circulation.

## Author contributions

All authors listed have made a substantial, direct, and intellectual contribution to the work and approved it for publication.

## Conflict of interest

The authors declare that the research was conducted in the absence of any commercial or financial relationships that could be construed as a potential conflict of interest.

## Publisher’s note

All claims expressed in this article are solely those of the authors and do not necessarily represent those of their affiliated organizations, or those of the publisher, the editors and the reviewers. Any product that may be evaluated in this article, or claim that may be made by its manufacturer, is not guaranteed or endorsed by the publisher.
